# Neuronal Chemosensation and Osmotic Stress Response Converge in the Regulation of *aqp-8* in *C. elegans*

**DOI:** 10.3389/fphys.2017.00380

**Published:** 2017-06-09

**Authors:** Carla Igual Gil, Mirko Jarius, Jens P. von Kries, Anne-Katrin Rohlfing

**Affiliations:** ^1^Zoophysiology, Institute for Biochemistry and Biology, University PotsdamPotsdam, Germany; ^2^Leibniz-Institut für Molekulare Pharmakologie (FMP)Berlin, Germany

**Keywords:** aquaporin, osmoregulation, osmotic stress, chemosensation, *C. elegans*

## Abstract

Aquaporins occupy an essential role in sustaining the salt/water balance in various cells types and tissues. Here, we present new insights into *aqp-8* expression and regulation in *Caenorhabditis elegans*. We show, that upon exposure to osmotic stress, *aqp-8* exhibits a distinct expression pattern within the excretory cell compared to other *C. elegans* aquaporins expressed. This expression is correlated to the osmolarity of the surrounding medium and can be activated physiologically by osmotic stress or genetically in mutants with constitutively active osmotic stress response. In addition, we found *aqp-8* expression to be constitutively active in the TRPV channel mutant *osm-9(ok1677)*. In a genome-wide RNAi screen we identified additional regulators of *aqp-8*. Many of these regulators are connected to chemosensation by the amphid neurons, e.g., *odr-10* and *gpa-6*, and act as suppressors of *aqp-8* expression. We postulate from our results, that *aqp-8* plays an important role in sustaining the salt/water balance during a secondary response to hyper-osmotic stress. Upon its activation *aqp-8* promotes vesicle docking to the lumen of the excretory cell and thereby enhances the ability to secrete water and transport osmotic active substances or waste products caused by protein damage. In summary, *aqp-8* expression and function is tightly regulated by a network consisting of the osmotic stress response, neuronal chemosensation as well as the response to protein damage. These new insights in maintaining the salt/water balance in *C. elegans* will help to reveal the complex homeostasis network preserved throughout species.

## Introduction

The control of intracellular osmolarity is an essential physiological property of life. In many animals, osmoregulation is a multi-organ comprising homoeostatic process that involves the body surface, the intestine, a secretory organ, and neuronal components. On a physiological level, aquaporins play an important role in maintaining the salt/water balance. Understanding aquaporin function and regulation helps to gain knowledge about this fundamental process. Our interest is to study the impact of aquaporins on the salt/water balance in *Caenorhabditis elegans*. As a well-established model organism, *C. elegans* is an ideal tool to investigate aquaporins and their involvement in adaptation to osmotic stress in a living multicellular organism. In *C. elegans*, the intestine, hypodermis, and excretory cell are involved in osmoregulation and several aquaporins (*aqp-1* to *-4, aqp-8*, and *aqp-10*) are expressed in these tissues (Huang et al., 2007). Genetic analyses revealed no significant differences in development, life span, fertility or mobility between aquaporin loss-of-function mutants and wild type animals under standard breeding conditions so far. Even though *aqp-2;aqp-3;aqp-4;aqp-8* quadruple mutants exhibit a sensitivity to hypo-osmotic stress (Huang et al., 2007). Here we concentrate on function and regulation of *aquaporin-8 (aqp-8)*, which is exclusively expressed in the excretory cell (Huang et al., 2007).

The hypodermis and the intestine adopt excretory/secretory functions in *C. elegans* and are important for osmoregulation and the osmotic stress response. These are the only tissues in direct contact to hyper-osmotic media. While the hypodermis is shielded to some extent by the cuticle, the intestine is directly exposed to salt-enriched food, protected only by a glycocalyx. The excretory cell has been presumed to be the *C. elegans* equivalent of excretory organs in other organisms and, thus important to maintain the salt/water balance and facilitate waste elimination (Nelson et al., 1983; Nelson and Riddle, 1984). The excretory cell body and nucleus are positioned next to the anterior portion of the terminal bulb of the pharynx (Nelson et al., 1983). Emerging from the cell body, pairwise extensions, the anterior and posterior excretory canals, are reaching throughout the entire body (**Figure 2A**). The apical membrane is lining a canal lumen and a complex network of canaliculi and vesicle surrounds the lumen of the excretory canals (Nelson et al., 1983). The small size of the excretory cell has prevented measurements of secretion/excretion but laser ablation of the excretory cell or the adjacent duct and pore cells of the excretory system results in fluid accumulation within the animals and subsequent death (Nelson and Riddle, 1984; Liégeois et al., 2007). Similar phenotypes have been observed in mutants with impaired tubulogenesis (Buechner et al., 1999; Mancuso et al., 2012).

*aqp-8* plays an important role in excretory cell development. Loss of *aqp-8* results in moderately shorter canals, whereas *aqp-8* overexpression causes widened canals (Khan et al., 2013). In tune with a role of *aqp-8* in excretory cell morphogenesis, AQP-8::GFP fusion protein localizes to vesicles and canaliculi surrounding the apical membrane of the excretory canal (Khan et al., 2013). The direct interaction between *aqp-8* and *erm-1*, the *C. elegans* ortholog of ERM (Ezrin-Radixin-Myosin) proteins, is necessary for the docking of vesicles and canaliculi to the canal lumen (Khan et al., 2013). It has been suggested that *aqp-8*-mediated water flow into the lumen together with vesicles docking to the luminal membrane contributes to anterior/posterior extension of the canal lumen during L1 stage outgrowth (Khan et al., 2013; Kolotuev et al., 2013). Water flow may also lead to a radial expansion of the lumen during later larval development (L2–L4 stage). The docking of vesicles and canaliculi is also triggered by moderate osmotic stress conditions (Khan et al., 2013; Kolotuev et al., 2013).

Only little is known about regulation of *aqp-8* expression. The POU homeobox transcription factor *ceh-6* (*C. elegans* homeobox-6) is necessary for *aqp-8* mRNA expression during development (Mah et al., 2007). *pros-1*, the *C. elegans* homolog of vertebrate Prox-1 transcription factor, has been shown to affect *aqp-8* indirectly, possibly by regulation of *ceh-6* (Kolotuev et al., 2013). The developmental Serial Analysis of Gene Expression (SAGE) profiles for *aqp-8* mRNA and transgenic lines expressing AQP-8::GFP fusion proteins showed that the *aqp-8* expression starts during L1 stage, reaches maximum levels at L4 stage and declines during adulthood (McKay et al., 2003; Mah et al., 2007). Hyper-osmotic stress causes an upregulation of *aqp-8* mRNA expression in adult animals (Rohlfing et al., 2010).

Hyper-osmotic stress causes severe protein damage and protein aggregation by macromolecular crowding within minutes to an hour after onset (Burkewitz et al., 2011). The activation of the hyper-osmotic stress response induces the transcription of the enzyme *glycerol-3-phosphate dehydrogenase-1* (*gpdh-1*) via the erythroid-Like transcription factors *elt-2* and *elt-3*, mainly in the intestine but also in the hypodermis (**Figure 5**; Lamitina et al., 2004; Rohlfing et al., 2010). Active *gpdh-1* produces high amounts of the protective osmolyte glycerol within the intestine and thereby generates an osmotic resistance that attenuates the macromolecular crowding. Whether the osmolyte glycerol is retained in the intestine or spread throughout the entire animal is not known. Potentially, it could be transported from the intestine into the body cavity by the aquaglyceroporine *aqp-1* to protect the whole animal. Several mutants constantly activate the osmotic stress response, including animals harboring mutations in collagens (*dpy-7/8/9/10*), mucin-like protein *osm-8*, and the potential notch-ligands *osm-7* and *osm-11* (Lamitina et al., 2006; Wheeler and Thomas, 2006; Rohlfing et al., 2010, 2011; Choe, 2013). All of these genes are either cuticle components or part of the hypodermal secretome into the cuticle. Mutations in these genes may affect an osmosensor connected to or imbedded in the cuticle (Rohlfing et al., 2011). The mucin-like protein *osmotic avoidance abnormal-8* (*osm-8*) is such a potential osmosensor. The mutants allele *osm-8(n1518)* does constitutively activate the osmotic stress response and might act similar to the osmosensor composed of the mucins Hkr1 and Msb2 in yeast (Tanaka et al., 2014). A side effect of the constitutive activation of the osmotic stress response is the accumulation of huge amounts of glycerol and the hyper-osmotic resistance of the mutant strains (Lamitina et al., 2004). This constitutive activation is dependent on the patch-related receptor *ptr-23* (**Figure 5**; Rohlfing et al., 2011). Interestingly, most of the *osmotic stress resistant* (*osr*) mutants, e.g., *osm-8(n1518)*, are also constitutively activating *aqp-8* mRNA expression under isotonic conditions (Rohlfing et al., 2010). The physiological (osmotic stress) and constitutive (*osr* mutants) activation of *aqp-8* mRNA expression makes *aqp-8* a target for the osmotic stress response.

Osmotic stress in the environment is also detected by chemosensory neurons in the amphid organ of *C. elegans*, which are required for chemotaxis and osmotic avoidance (Bargmann, 2006). Animals defective in chemosensation by the amphid neurons fail to avoid regions of osmotic stress. For example, the TRPV channel mutants *osm-9(ok1677)* does lack osmosensing by the amphid neurons and fails to avoid hyper-osmotic conditions (Bargmann, 2006). These neuronal defective osmotic avoidance mutants are not activating *gpdh-1* and are thus not osmotic resistant. A cross talk between the osmotic stress response triggered by the cuticle and the osmotic avoidance triggered by the sensory neurons has not yet been observed.

Here, we investigate the function of *aqp-8* in the adult excretory cell. To investigate how *aqp-8* is regulated and whether the regulatory pathway is directly or indirectly connected to the osmotic stress response, we measured the *aqp-8* mRNA expression and protein levels under various osmotic stress conditions and in osmotic stress resistant mutants. A genome wide RNAi screen was performed to search for enhancers and suppressors of *aqp-8* expression. Our work established a connection between osmotic stress response and osmosensing pathways in the amphid neurons. This finding raises the intriguing possibility that neuronal osmosensing has not only a behavioral output but also a direct impact on the physiological response to osmotic conditions in the environment via regulating *aqp-8* expression.

## Results

**Figure 1:**
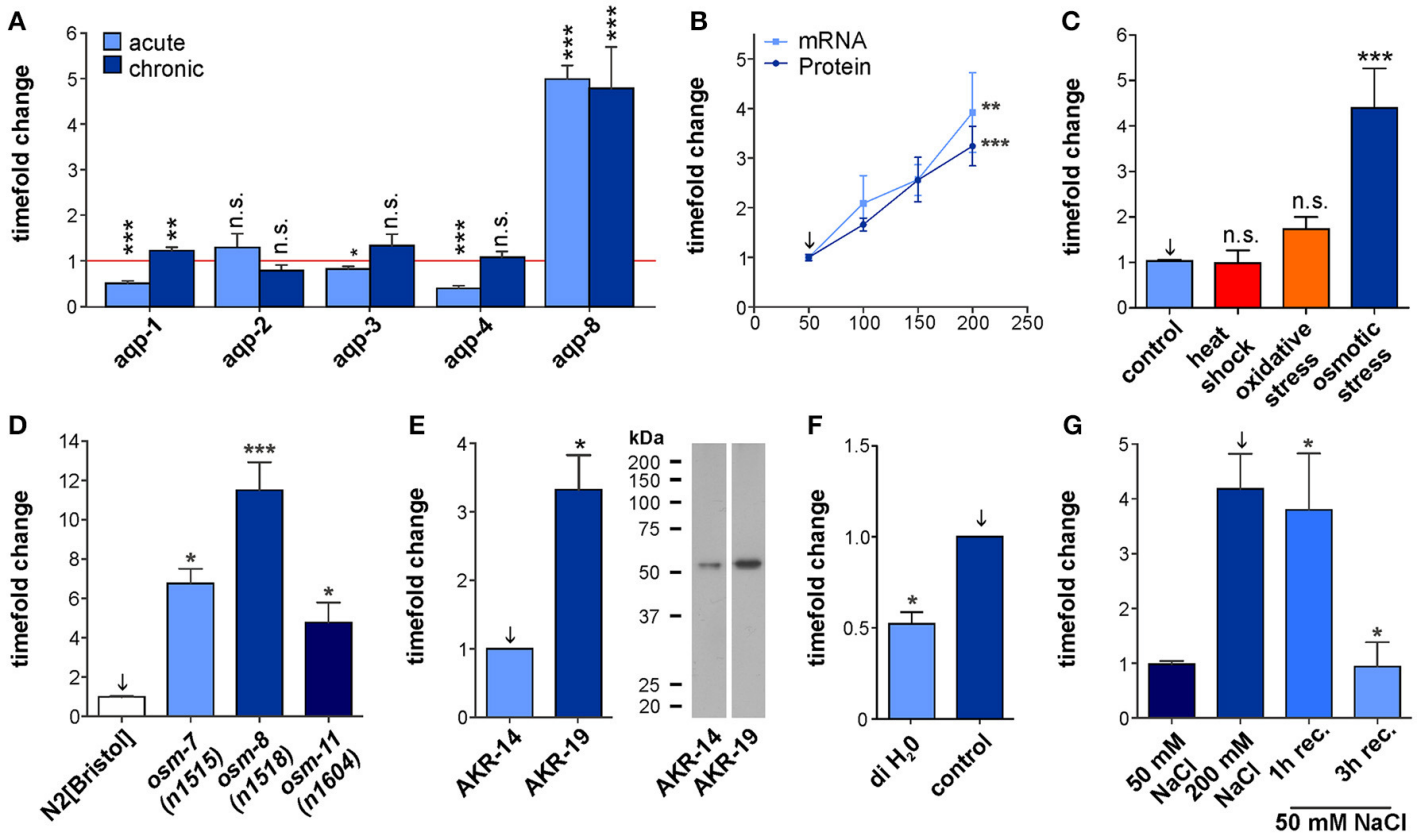
Characterization of *aqp-8* expression. **(A)** Effects of acute and chronic osmotic stress on the various aquaporines expressed in the intestine and the excretory cell. The amount of mRNA was measured by qPCR techniques in N2[BRISTOL] background. Student's *t*-test was performed against isosmotic control conditions set to one of each aquaporine. *N* > 4 **(B)** qPCR (light blue square; *N* > 5) and western blot (dark blue circle; *N* > 7) quantification of *aqp-8* expression levels under chronic osmotic stress conditions in a N2[BRISTOL] background. Student's *t*-tests were performed between 50 mM control (arrow) and 200 mM stress conditions. **(C)** qPCR quantification of the effect of different stress conditions onto the *aqp-8* mRNA expression in N2[BRISTOL]. Student's *t*-tests were performed between 50 mM NaCl (arrow) and the different stressors. *N* > 3 **(D)** qPCR analysis of the of *aqp-8* mRNA expression levels in *osmotic stress resistant* strains on 50 mM NaCl compared to wild type N2[BRISTOL]. Student's *t*-tests were performed between N2[BRISTOL] (arrow) and the *osm* strains. *N* > 4 **(E)** Western blot analysis and example of the of AQP-8 protein concentration in AKR-19 *osm-8(n1518);aqp-8::GFP* on 50 mM NaCl compared to wild type AKR-14 +/+;*aqp-8*::GFP. A student's *t*-test was performed between both samples; *N* = 6 **(F)** qPCR analysis of the *aqp-8* mRNA expression in N2[BRISTOL] under control and after 1 h exposure to hypo osmotic conditions, distilled water. Student's *t*-test were performed between the two conditions. *N* = 3 **(G)** qPCR analysis of the recovery of *aqp-8* mRNA expression on 50 mM NaCl NGM from chronic osmotic stress conditions (200 mM) in an N2[BRISTOL] background. Student's *t*-tests were performed between 200 mM NaCl chronic stress (arrow) and the recovery conditions; *N* > 4. All qPCR expression levels were normalized to *act-2* (actin). Western blot results were normalized to whole protein. All values are indicated relative to control conditions (set to 1). ^*^p < 0.05; ^**^p < 0.01; ^***^p < 0.001; n.s., not significant.

To test whether the aquaporines *aqp-1* to *-4* and *aqp-8* expressed within the tissues important for osmoregulation (Huang et al., 2007) are also affected by hyper-osmotic stress conditions on an expressional level, we measured their mRNA levels using qRT-PCR techniques (Figure 1A). To induce acute hyper-osmotic stress, synchronized populations of wild type *C. elegans* were exposed for 6 h to 200 mM NaCl in NGM at the first day of adulthood. Chronic stress conditions were generated by raising synchronized wild type animals on NGM containing 200 mM NaCl from L1 until the first day of adulthood. This treatment induces the osmotic stress response while still allowing the animals to develop and reproduce normally with a standard life span. *aqp-8* mRNA levels were significantly elevated under both acute and chronic hyper-osmotic stress conditions, as could be expected from previously performed microarrays which identified *aqp-8* as an osmotically regulated gene (ORGs), significantly up-regulated under hyper-osmotic stress conditions and in mutants with constitutive active osmotic stress response (Figures 1A,D; Rohlfing et al., 2010). Acute hyper-osmotic stress at day 1 of adulthood affected the expression of all tested aquaporines, except for *aqp-2*. In contrast to *aqp-8, aqp-1, aqp-3*, and *aqp-4* were significantly down regulated compared to control conditions (Figure 1A). Under chronic hyper-osmotic stress *aqp-1* was slightly but significantly up-regulated (Figure 1A) while *aqp-2, aqp-3*, and *aqp-4* were not significantly altered compared to control conditions. Hence, *aqp-8* exhibits the strongest and persistent osmo-response and is regulated differently to the other aquaporins by hyper-osmotic stress.

### *aqp-8* mRNA expression and protein concentration is closely linked to the osmotic milieu

To characterize the osmosensitive response of *aqp-8* expression more precisely, we performed qRT-PCR on animals raised on four different NaCl concentrations chronically applied. This experiment showed a linear and significant increase in *aqp-8* mRNA levels in correlation to the applied salt concentration (Figure 1B). The results of a western blot analysis confirmed this mRNA results. Protein levels increased in the same degree as the mRNA concentration (Figure 1B, Supplementary Figure 1D). In the absence of a suitable AQP-8 antibody, we used the strain AKR-14 +/+; akaIs2 [K02G10.7(translational)::GFP + pCeh361]. The strain does express a AQP-8::GFP fusion protein under the control of the endogenous promotor for *aqp-8*. We measured the changes in the AQP-8::GFP fusion protein concentrations using a GFP antibody. The antibody was evaluated for this use and we were able to detect a single band with the predicted molecular weight of 54.45 kDa (Supplementary Figures 1A–C). In summary, we uncovered a linear correlation between *aqp-8* mRNA and protein abundance with the surrounding osmolarity (Figure 1B).

### *aqp*-8 expression is selectively induced by the hyper-osmotic stress response

To test whether the *aqp-8* mRNA expression is specifically regulated by osmotic stress conditions, we assayed its expression in animals that were heat-shocked (1/2 h at 35°C) or underwent oxidative stress (1 h, 200 mM paraquat in NGM). However, only acute and chronic osmotic stress conditions significantly changed the abundance of *aqp-8* mRNA (Figure 1C).

### In the osr mutants *osm-7(n1518), osm-8(n1518)*, and *osm-11(n1604)* the osmotic stress response is constitutively active under isotonic conditions

Loss of any of these three *osm* proteins in the cuticle causes an activation of the cuticular osmotic stress response pathway (Rohlfing et al., 2011). Based on a microarray, we had previously identified increased *aqp-8* mRNA levels in the three *osr* mutants even under isotonic conditions (Rohlfing et al., 2010). We now confirmed this observation by qRT-PCR (Figure 1D). Similarly, AQP-8 protein levels were elevated in the *osm-8* strain (Figure 1E). Again transgenic lines expressing AQP-8::GFP were used to measure AQP-8 protein levels using the established GFP antibody. Taken together, these results demonstrate that *aqp-8* expression is specifically induced by the hyper-osmotic stress response and that activation can occur physiologically (hyper-osmotic stress) or constitutively (mutant).

### Detection of the isoform b of *aqp-8* K02H10.7

Since *aqp-8* has two predicted isoforms, K02H10.7a (1022 nt) and K02H10.7b (1134 nt; Supplementary Figure 2A), we also tested by qRT-PCR whether the truncated isoform a is present and affected by the osmotic stress response. However, agarose gel analyses of PCR amplified cDNA samples from wild type strain N2[Bristol] and *osm-8(n1518)* animals (Supplementary Figure 2B) revealed that only the full-length isoform b was present in our samples, Subsequent sensitive melting curve analysis confirmed this result (Supplementary Figure 2C). In coherence with the above described constitutive activation of *aqp-8* in an *osm-8(n1518)* background, the amount of PCR product in the *osm-8(n1518)* sample is visibly increased compared to N2[BRISTOL] by equal amounts of cDNA used in the PCR reaction.

### The hypo-osmotic stress reduces *aqp-8* expression

To test whether hypo-osmotic stress has an opposite effect on *aqp-8* mRNA expression, we placed young adult wild type animals, reared at control conditions (50 mM NaCl in NGM), in deionized water (60 min treatment). In line with a role of *aqp-8* regulation in osmotic stress response, acute exposure to hypo-osmotic conditions induced a significant decrease in *aqp-8* mRNA levels within 1 h upon treatment (Figure 1F). This finding was consistent with the regulation of *aqp-8* upon recovery from hyper-osmotic stress (Figure 1G). Within 3 h of recovery, *aqp-8* mRNA levels had decreased to control levels. Hence, *aqp-8* expression is negatively regulated under different hypo-osmotic stress conditions.

### The excretory cell adapts to osmotic stress conditions

**Figure 2:**
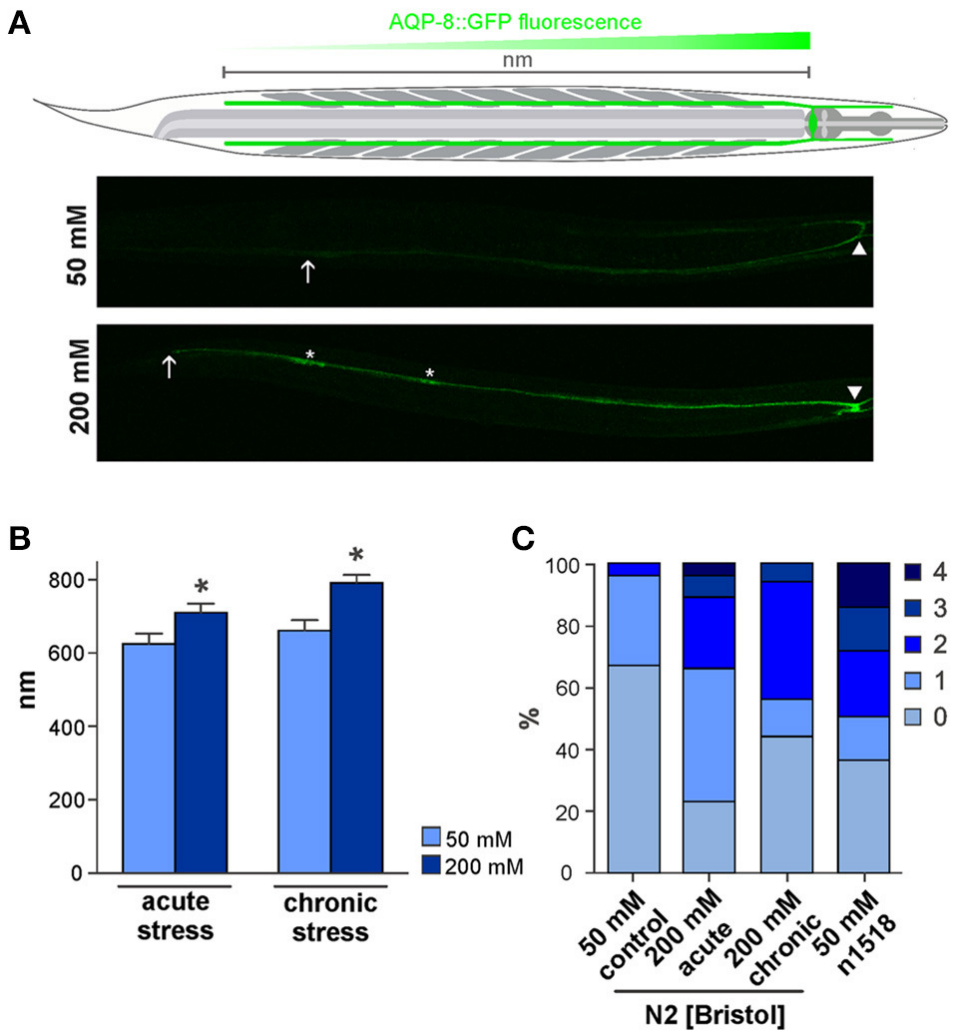
AQP-8 protein expression throughout the excretory canal and *aqp-8* isoform analysis. **(A)** Schema of the *C. elegans* and the excretory cell. Example of the AKR-14 +/+;*aqp-8*::GFP expression in the posterior excretory canal under control and chronic stress conditions. The arrows indicate the maximum spreading of the signal, starting from cell soma (arrow head) throughout to the posterior canal (arrow). Varicosities are marked by asterisk. **(B)** Analysis of the AKR-14 +/+;*aqp-8*::GFP fluorescence pattern after 8 h acute and chronic 200 mM NaCl stress conditions in the excretory cell compared to 50 mM NaCl control conditions. The spreading of the GFP signal from the cell soma throughout the posterior excretory canal was measured and plotted. Student's *t*-tests were performed relative to control conditions; N>16 (^*^p < 0.05). **(C)** Number of varicosities visible in one posterior excretory canal per animal. The number of canals with varicosities is significantly lower under control conditions compared to acute (*p* = 0.0008) and chronic (*p* = 0.0070) stress conditions. There are significantly more varicosities in an *osm-8(n1518)* (*p* = 0.0007) background compared to wild type animals even under isotonic conditions, as determined by Students *t*-test. *N* > 14.

To investigate the effect of osmotic stress conditions on the localization of AQP-8 in the adult excretory cell, we characterized AQP-8::GFP localization using standard fluorescence microscope techniques. Under normal osmolarity conditions, AQP-8::GFP localized within the excretory cell soma and throughout the entire anterior excretory canal. Within the posterior excretory canal, the signal intensity decreased from the soma toward the tail region (Figure 2A), although the canal is fully extended throughout the whole body in adults (Khan et al., 2013; Kolotuev et al., 2013). In most animals, the signal was only visible along ~80% of the length of the posterior canal. Under acute hyper-osmotic stress conditions (8 h, 200 mM NaCl in NGM), the AQP-8::GFP signal expanded from the soma throughout the posterior canal (Figures 2A,B). Animals raised from L1 stage under chronic hyper-osmotic stress conditions (200 mM NaCl in NGM), showed a bright fluorescence signal within the entire excretory canal (Figure 2B). The body length was not significantly different between control and treated animals and therefore was not taken into account in our measurements (Supplementary Figure 3). In summary, the expansion of the AQP-8::GFP signal toward the tip of the canal was significantly increased by acute and chronic stress conditions (Figure 2B).

When larvae are exposed to hyper-osmotic stress conditions, pearl-string like varicosities have been reported to appear during excretory cell development (Khan et al., 2013; Kolotuev et al., 2013). These swellings represent extensions of the endoplasmic reticulum and Golgi apparatus into the posterior canal, which supplies vacuoles and canaliculi (Kolotuev et al., 2013). Similarly, we observed the appearance of AQP-8::GFP positive varicosities in the posterior canal (Figure 2A, asterisk). In our experiments, 33% of control animals developed one or rarely two varicosities within the posterior canal (Figure 2C). This number increased significantly to 77% (*p* = 0.0003) with one to four varicosities per canal when animals were exposed to acute osmotic stress conditions (8 h/200 mM NaCl). The number of varicosities in chronically treated animals (starting from L1) was lower but still significantly elevated (56%; *p* = 0.0113) compared to control conditions (Figure 2C). In an *osm-8(n1518)* background the amount of varicosities is also significantly increased (64%; *p* = 0.0007) under isotonic growth conditions (Figure 2C), which indicates an involvement of the osmotic stress response pathway in the formation of varicosities. Taken together, these results suggest that the excretory cell, when challenged with osmotic stress, uses its entire length and organelle supply for osmoregulation.

### Glycerol accumulation is not the trigger for *aqp-8* mRNA expression

To further characterize how the osmotic stress response pathway and *aqp-8* activation are linked, we functionally tested the involvement of glycerol synthesis in this regulation. *gpdh-1* expression is activated by the transcription factor *elt-2* within the intestine and, to a negligible degree, by *elt-3* within the hypodermis (Rohlfing et al., 2011). *gpdh-1* is the final target gene in the osmotic response pathway and produces the protective osmolyte glycerol (**Figure 5**; Rohlfing et al., 2011). Conversely, the patch-related receptor *ptr-23* is an upstream suppressor of the signal cascade, possibly receiving direct input from an osmosensor in the cuticle (**Figure 5**; Rohlfing et al., 2011). An intermediate pathway has not been uncovered yet. We therefore asked, whether *aqp-8* and *gpdh-1* could partially share a common signaling pathway downstream of *ptr-23*, or whether the internal glycerol concentration itself could be a signal for *aqp-8* expression.

**Figure 3:**
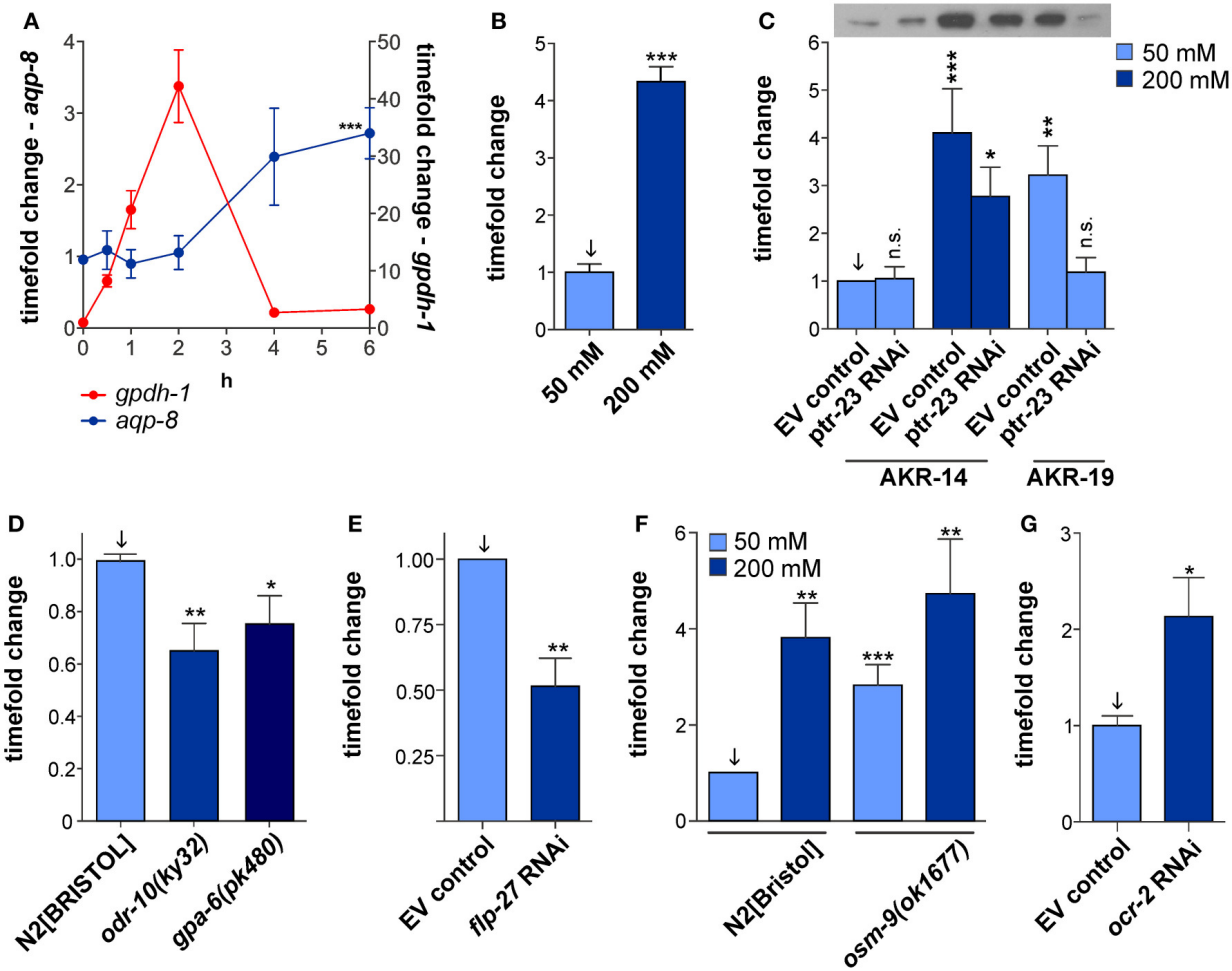
Possible regulatory pathways of *aqp-8* expression. **(A)** Comparison of *aqp-8* and *gpdh-1* mRNA expression pattern after acute osmotic stress onset in a N2[BRISTOL] background, *gpdh-1* (red circle, right y-axis) and *aqp-8* (blue circle, left y-axis). The 0 h control was set to 1. A Student's *t*-test for *aqp-8* induction was performed between 6 h 50 mM (Supplementary Figure 4) and 6 h 200 mM stress conditions. *N* > 4 **(B)** qPCR analysis of the *aqp-8* mRNA expression under chronic osmotic stress conditions in an *gpdh-1(ok1558)* mutant background. A Student's *t*-test were performed between 50 mM NaCl control condition (light blue) and chronic osmotic stress conditions (dark blue); *N* = 6. **(C)** Western blot analysis of the *ptr-23* RNAi effect on AQP-8 protein concentrations in ARK-14;+/+;*aqp-8*::GFP and AKR-19*;osm-8(n1518);aqp-8*::GFP background. A sample western blot is shown above the graph, loaded in the same order as the subjacent graph. Student's *t*-tests were performed compared to AKR-14 EV control conditions (set to 1); *N* > 4 **(D)** qPCR analysis of the of *aqp-8* mRNA expression levels in *osr-10(ky32)* and *gpa-6(ok480)* strains on 50 mM NaCl compared to wild type N2[BRISTOL]. Student's *t*-tests were performed between N2[BRISTOL] (arrow) and the two strains. *N* > 6 **(E)** Western blot analysis of *flp-27* RNAi impact on AQP-8::GFP expression. A Student's *t*-test was performed against wild type control (arrow). *N* = 4 **(F)** qPCR analysis of the of *aqp-8* mRNA expression levels in *osm-9(ok1677)* compared to wild type N2[BRISTOL] under control and osmotic stress conditions. Student's *t*-tests were performed and results compared to wild type control (arrow). N>6. **(G)** Western blot analysis of *ocr-2* RNAi impact on AQP-8::GFP expression. A Student's *t*-test was performed against wild type control (arrow). *N* = 7. All qPCR expression levels were normalized to *act-2* (actin). Western blot results were normalized to whole protein. All values are indicated relative to control conditions (set to 1). ^*^p < 0.05; ^**^p < 0.01; ^***^p < 0.001; n.s., not significant.

To address this question, we correlated *gpdh-1* expression and onset glycerol accumulation to *aqp-8* activation. Under moderate osmotic stress condition (200 mM NaCl in NGM) applied at day 1 of adulthood, *gpdh-1* mRNA levels were elevated within half an hour after the onset of the moderate osmotic stress reaching maximum levels after 2 h (Figure 3A). *gpdh-1* and glycerol levels have been shown to be tightly linked, glycerol levels start to rise as soon as the enzyme is present (Lamitina et al., 2004). Under our test conditions the osmolyte glycerol should have started to accumulate between half an hour to one hour after the onset of the stress. The *aqp-8* mRNA levels started to increase after a lag period of ~3 h. After 6 h, a significant increase in *aqp-8* mRNA was observed compared to control conditions (50 mM NaCl; Figure 3A, Supplementary Figure 4). At the onset of *aqp-8* mRNA expression, a significant amount of glycerol should be present. Therefore, glycerol accumulation could be a possible trigger for *aqp-8* mRNA expression.

Next, we tested whether the inhibition of glycerol accumulation would affect *aqp-8* mRNA expression. We used the *gpdh-1* loss-of-function allele *ok1558* and applied acute osmotic stress (6 h, 200 mM NaCl in NGM) at day 1 of adulthood. The loss of glycerol accumulation in the mutant did not impair with a significant activation of *aqp-8* mRNA expression (Figure 3B). In correspondence to this results, the RNAi knock-down of the *gpdh-1* transcription factor *elt-2* did not affect the constitutive activation *aqp-8* expression the *osm-8(n1518)* mutant (Supplementary Figure 5). Taken together, glycerol accumulation does not seem to be the trigger for *aqp-8* expression.

### *aqp-8* expression can be activated by more than one pathway

To investigate the direct impact of *ptr-23* on AQP-8 protein concentrations, we performed *ptr-23* RNAi knock-down in wild type and *osm-8(n1518)* backgrounds. Western blot analysis revealed that the constitutive high AQP-8 protein concentration in *osm-8(n1518)* mutants is reduced to normal values upon loss of *ptr-23* via RNAi knock-down (Figure 3C). However, the physiological-induced increase in AQP-8 protein concentration by hyper-osmotic stress in wild type animals was only partially affected by *ptr-23* RNAi knock-down (Figure 3C). Hence, additional pathways, besides the *ptr-23*-mediated activation, could be present to induce of *aqp-8* expression.

### A genome-wide RNAi screen reveals additional pathways involved in *aqp-8* activation

To identify *ptr-23*-independent pathways involved in AQP-8 regulation, we performed a genome-wide RNAi screen using the established Ahringer RNAi library on our AQP-8::GFP reporter strain AKR-14 grown under standard conditions (NGM, 50 mM NaCl). Changes in AQP-8::GFP transgene expression levels were monitored using a standard fluorescence stereomicroscope.

In total, we identified 687 genes with an effect on AQP-8::GFP expression out of 19,762 tested genes. Many of these genes affect basic cellular processes including ribosome function or metabolism. Surprisingly, 63 genes (9.17% of all hits) were connected to neuronal function or neural development. Subsequently, the RNAi efficacy for these most interesting candidates was re-examined and quantified by Western blot. AQP-8 protein expression was reduced by at least 20% upon loss of 17 genes that are connected to neuronal function tested in the secondary screen while the loss of another neuronal gene activated AQP-8 expression by 1.5-fold (Supplementary Table 1). The list includes among others various G-protein coupled receptors, two FMRF-like peptides, a glutamate gated chloride channel and an ionotropic glutamate receptor. Since neuronally-expressed genes have previously been described to be refractory to RNAi (Timmons et al., 2001), we also analyzed the *aqp-8* mRNA expression in corresponding mutants of some interesting candidates, that had failed to pass the secondary RNAi screen. This approach further confirmed the seven transmembrane G-protein coupled olfactory receptor gene *odr-10* (Figure 3D). *odr-10* has been linked to chemosensation by amphid neurons (Sengupta et al., 1996; Troemel et al., 1997; Zhang et al., 1997). Using qRT-PCR techniques in the *odr-10* mutant allele *ky32*, we detected a significant 0.45-fold reduction of *aqp-8* mRNA expression (Figure 3D). The secondary screen also identified the G-protein encoding gene *gpa-6*, which may function in amphid neuron chemosensation and is co-expressed with *odr-10* (Lans et al., 2004). Western blot analysis revealed a reduction in AQP-8 levels by 45% upon RNAi knock-down of *gpa-6* (Supplementary Table 1). We confirmed the effect of *gpa-6* by measuring the *aqp-8* mRNA concentration in the mutant *gpa-6(pk480)*. The qRT-PCR results revealed a significant reduction of *aqp-8* expression (Figure 3D).

Two other interesting candidates from our genome wide RNAi screen are the FMRF-like peptides *flp-25* and *flp-27*. Especially *flp-27* RNAi significantly suppresses *aqp-8* expression levels compared to control levels (Figure 3E). In the kidney of vertebrates aquaporin location and expression are regulated by the peptide hormone vasopressin (Borgnia et al., 1999). *C. elegans* exhibits a vasopressin homolog called nematocin (*ntc-1*) and two nematocin receptors *ntr-1* and *ntr-2* (Beets et al., 2012 and Garrison et al., 2012). Although, none of these receptors is expressed in the excretory cell, we tested nematocin for a regulatory effect on *aqp-8* expression. RNAi against nematocin failed to affect *aqp-8* RNA expression under isotonic and acute hyper-osmotic stress conditions, but did alter the expression of other *C. elegans* aquaporins which are susceptible to *ntr-1* and *ntr-2* signaling under the mentioned conditions (data not shown). The FMRF-like peptide *flp-27* may have a modulatory function within the neuronal network or it could have taken over the role of nematocin in the excretory cell and may constitute the connection between neuronal osmosensing and tissue specific effects. All four candidates, *odr-10, gpa-6, flp-25*, and *flp-27* establish a connection between osmosensing in the amphid neurons and the physiological response to osmotic stress.

### *osm-9* is another neuronal expressed candidate for controlling *aqp-8* expression

*osm-9* is a member of the TRPV channel family and plays major roles in transduction and regulation of signals in several sensory neurons. The channel is important for processes such as water-soluble chemotaxis, volatile chemotaxis, and osmotic avoidance (Colbert et al., 1997; Bargmann, 2006). However, contrary to other *osm* genes, *osm-9* has not been associated with the osmotic stress response or glycerol accumulation. Recently, the *osm-9* mutant allele *ok1677* has been shown to promote proteostasis and induce the expression of genes that manage protein damage (Lee et al., 2016). Since protein damage occurs in *C. elegans* upon exposure to osmotic stress conditions (Burkewitz et al., 2011), *osm-9* might be linked to another branch of the osmotic stress response independent of glycerol activation. *osm-9* was not identified in our genome wide screen, but *osm-9* is co-expressed with *gpa-6* and *odr-10* in some amphid neurons and all three genes potentially encode components of a common signaling pathway regulating *aqp-8* expression (Bargmann, 2006). Furthermore, its role in osmosensing and proteostasis described above made *osm-9* an even more interesting candidate. *osm-9* RNAi proved to be refractory. Hence, we tested *aqp-8* mRNA levels in an *osm-9(ok1677)* background and detected a 2.81-fold increase under isotonic conditions (Figure 3F). For proper localization in the sensory cilia of the amphid neurons and chemosensation, the OSM-9 TRPV channel requires co-expression of the TRPV channel OCR-2. OSM-9 forms a heterotetramer with OCR-2, which functions in chemosensation, but not in other functions such as e.g., sensory adaptation (Tobin et al., 2002). Consistent with an involvement of OCR-2 in osmoregulation, the RNAi knock-down of *ocr-2* in the AKR-14 background caused a significant AQP-8 protein overexpression similar to *osm-9(ok1677)* overexpression (Figure 3G). These results provide further evidence for an involvement of the OSM-9/OCR-2 TRPV channel in *aqp-8* regulation. Further experiments have to be performed to explore the cross talk between *osm-9/ocr-2, gpa-6* and *odr-10* but potentially all four proteins could function as components of a consecutive pathway in the AWA sensory neuron, in which they are co-expressed.

## Discussion

Maintaining intracellular homeostasis is important to ensure the proper function of all physiological processes, e.g., the proteostasis network. To avoid loss of important physiological functions or macromolecular crowding, single cells, and organs have developed several mechanisms to protect themselves from strong fluctuations in the osmolarity. Aquaporins are some of the main players in maintaining homeostasis. Osmotic stress from the surrounding media or the environment challenges this fragile balance and prompts a fast response to avoid cellular damage. Beside the production or uptake of protective osmolytes such as glycerol, sucrose, some amino acids or even urea, controlling aquaporin function is an important part of this response. Several parallel pathways, like osmotic avoidance or osmotic stress response, tightly control the salt and water balance in *C. elegans*. The role of aquaporins in this process is poorly investigated. Therefore, we studied the regulation of aquaporins expressed in the intestine, hypodermis and excretory cell of *C. elegans*. These are the main in osmoregulatory tissues of *C. elegans*.

Most aquaporins expressed in the osmoregulatory tissues, except *aqp-2*, are affected by acute hyper-osmotic stress in the environment (Figure 1A). *aqp-2* is not regulated on a transcriptional level, but could still be regulated upon stress on a translational level or by protein degradation. Retention away from the cellular membrane might also play a role, similar to human AQP-2 in the kidney. It should be mentioned here that the nomenclature of *C. elegans* aquaporins is not congruent with the human/vertebrate nomenclature and *C. elegans*, e.g., *aqp-2* is not a homolog of human AQP-2. Function and regulation of *aqp-2* will need to be further investigated, to rule out alternative regulatory mechanisms. In contrast, *aqp-1, aqp-3*, and *aqp-4* were significantly down regulated under acute hyper-osmotic stress compared to control conditions (Figure 1A). This decrease in aquaporin levels in the intestine and in the excretory cell helps to retain water within the animal, which otherwise would be rapidly lost by migration along the existing osmotic gradient. In contrast, reduction in aquaglyceroporins should help to accumulate the protective osmolyte glycerol within the animal. Under chronic stress glycerol accumulates to great amounts (Lamitina et al., 2004). This osmotic stress response protects the animals. Under chronic stress conditions *aqp-1* is just slightly but significantly up-regulated and might help to distribute the protective glycerol throughout the animal after a critical concentration is reached inside the intestine. The active protection machinery probably abolished the acute regulatory effects on *aqp-3* and *aqp-4* and the aquaporins can resume their regular functions in the *C. elegans* physiology (Figure 1A).

In contrast to the other four aquaporins, *aqp-8* mRNA and protein concentrations were specifically and significantly elevated upon the onset of hyper-omotic stress conditions and the transcription remained activated to the same extent as long as the stress situation prevailed (Figure 1A). *aqp-8* expression was also induced by constitutive activation of the osmotic stress response in osmotic resistant mutants (Figure 1D). These results suggest an important function of *aqp-8* in the acute and chronic osmotic stress response, which requires a constant high concentration of the aquaporin. Because of this unique expression pattern, we focused our further work on *aqp-8*.

**Figure 4:**
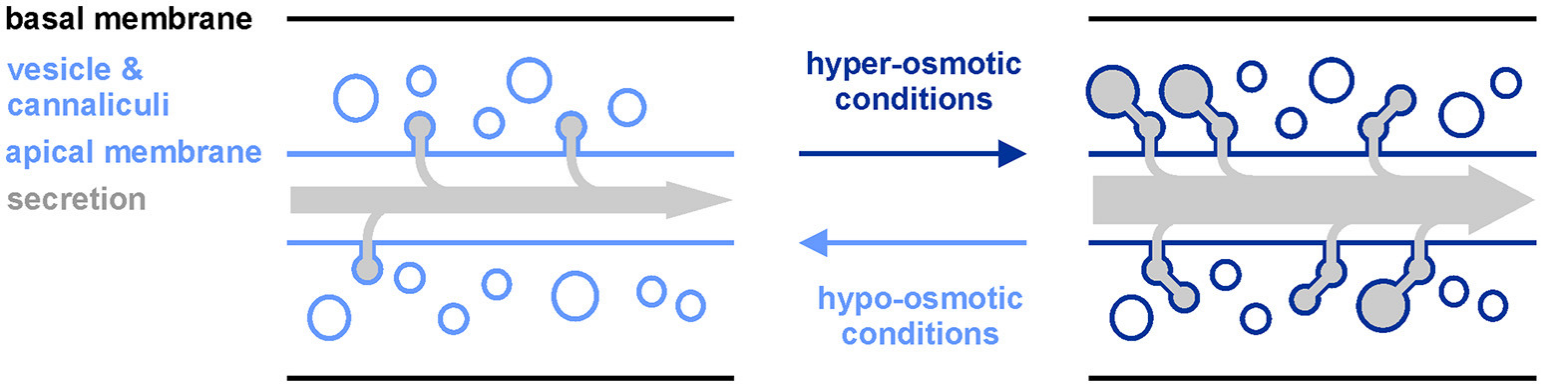
Model of *aqp-8* induced changes in excretory canal network upon alterations in the surrounding osmolarity. Upon exposure to hyper-osmotic conditions, *aqp-8* expression is activated and enables more vesicles and canaliculi to attach to the excretory canal lumen. Thereby, excretes from vesicles are delivered into the canal lumen and the surface of the lumen is expanded to facilitate further secretion/excretion via the canal membrane. Hypo-osmotic conditions should have the reverse effect on the excretory canal network and the secretion/excretion capacity.

The major function of AQP-8 might be to enable vesicle docking to the excretory cell lumen together with ERM-1, as has been demonstrated during excretory cell development (Figure 4; Khan et al., 2013; Kolotuev et al., 2013). The water or glycerol transport activity of the expressed functional full-length AQP-8 isoform b could support active salt transport into the lumen and helps to flush secrete out of the excretory canal (Supplementary Figures 2A,B). But, as water and glycerol loss is critical under hyper-osmotic stress conditions, it is maybe subsidiary role of AQP-8 compared to in vesicle docking. New vesicles could segregate from the golgi apparatus in the varicosities, which appear during stress conditions. Expanding the vesicle and canaliculi network increases the luminal membrane surface and might be needed to shuttle transport protein into the membrane. We could explain the constant high expression of *aqp-8* by the demand for AQP-8 protein for newly produced vesicles, as long as the stress prevails. Our data show a fast response on the *aqp-8* mRNA expression levels under hypo-osmotic stress conditions or during recovery (Figures 1F,G). Within an hour the concentration is significantly decreased. Consistent with our theory this fast response should help to maintain the intracellular homeostasis by retaining osmotically active substances and water within the body of the animal (Figure 4).

We also measured a time lag between onset of stress and *aqp-8* induction (Figure 3A). These results suggest that *aqp-8* is not part of the primary response, like *gpdh-1* activation. However, *aqp-8* might be activated during a secondary response upon a specific trigger. This trigger is not glycerol, as we were able to demonstrate, but it may be critical amounts of osmotic active substances or the accumulation of waste products from damaged cell components. Hyper-osmotic stress leads to macromolecular crowding, as protein aggregation and damage occur rapidly after the onset of hyper-osmotic stress (Burkewitz et al., 2011). Toxic waste products and osmotic active substances could be secreted from the excretory cell by the secretory vesicles and the enlarged membrane surface of the extended luminal network. This result would be in line with our finding that the intensity of the osmotic stress is correlated with the amount of AQP-8. The stronger the stress, the more osmotically active substances and waste products have to be secreted, and the more vesicles are needed (Figure 4). This also affects the activation state of the excretory cell itself. This has been demonstrated by the extension of the AQP-8::GFP signal throughout the posterior excretory canal and the appearance of varicosities upon onset of hyper-osmotic stress in the environment or by constitutive activation of the osmotic stress response (Figures 2A,C).

Interestingly, recent findings have shown an improved proteostasis in the mutant *osm-9(ok1677)* under osmotic stress conditions (Lee et al., 2016). The mutation constantly activates genes, which normally respond to protein damage. Furthermore, *osm-9* is one of the key proteins in chemosensation and osmotic avoidance by the amphid neurons. *osm-9(ok1677)* mutants are osmotic avoidance abnormal and thereby fail to avoid of high levels of sodium chloride in the surrounding environment. But *osm-9* and *osm-9* mutants do not induces glycerol accumulation and osmotic stress resistance. We found *osm-9(ok1677)* to constitutively activate *aqp-8* expression under isotonic conditions using RT-qPCR techniques (Figure 3F). *osm-9* encodes a TRPV channel protein (Colbert et al., 1997). In most neurons the protein OSM-9 forms a hetero-tetramer with OCR-2, another TRPV protein, to form a functional channel protein localized to the cilium (Tobin et al., 2002). Hence, we could show that RNAi knock-down of the gene *ocr-2* also constitutively induced *aqp-8* mRNA expression (Figure 3G). AQP-8 is not included on the list of putative proteostasis genes, which Lee et al. (2016) found to be up-regulated in the allele *ok1677*, as *aqp-8* has not been previously connected to macromolecular crowding or protein damage. This finding further supports our hypothesis that AQP-8 could be important for the secretion of osmotic active substances and waste products during osmotic stress. AQP-8 is, to our knowledge, the first protein on which the osmotic stress response pathway converges with a neuronal response to control expression levels.

**Figure 5:**
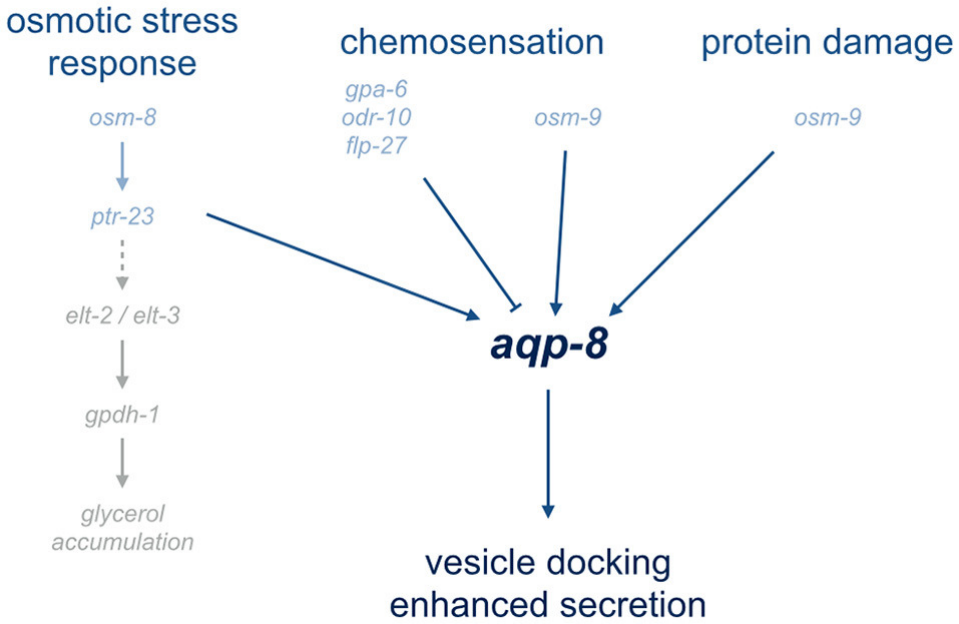
Overview of pathways involved in aqp-8 regulation. The overview illustrates the signaling pathways involved in aqp-8 regulation. Genes connected to or involved in the diverse pathways are depict in light blue. Represented in light gray are the identified components of osmotic stress response pathway leading to glycerol accumulation. A dashed line indicates the unknown components of the gpdh-1 activation pathway.

Our genome-wide RNAi screen further confirmed the participation of the amphid neurons in *aqp-8* regulation. The neuronal expressed G-protein coupled receptor ODR-10 and the G-protein GPA-6 emerged from the screen. They are reducing the *aqp-8* expression under standard growth conditions. Therefore, neuronal chemosensation plays a role in *aqp-8* regulation at least under isotonic conditions. Furthermore, ODR-10 and GPA-6 are possible interaction partners of the OSM-9/OCR-2 TRPV channel in the some amphid neurons, e.g., the AWA neurons (Sengupta et al., 1996; Bargmann, 2006). The FMRF-like-peptide FLP-27 is one candidate for a neuronally derived regulator or modulator of *aqp-8* expression downstream from the actual osmosensing process. FLP-27 could be the missing link between sensory neurons and the tissue specific effects in the excretory cell or neuronal modulator. Further experiments have to be performed to verify these hypotheses, for example by identification and location of FLP-27 specific receptors. All five proteins have an impact on *aqp-8* regulation, although diametrically opposed (Figure 5). Whether and how these proteins are part of one neuronal signaling pathway or are independent from each other has to be further analyzed as well as their connection to the other neuronal genes identified in our screen (Supplementary Table 1). These experiments helped to identify the neuronal network and signaling pathways involved in coping with acute osmotic stress conditions by activating the excretory cell. Further, experiments have to be performed to investigate the connection and a possible cross talk between the classical osmotic stress response, chemosensation, and protein damage response.

To summarize, we found a unique expression pattern for *aqp-8* upon hyper-osmotic stress compared to other aquaporins expressed in osmoregulatory tissues. Under hyper-osmotic stress the main function of *aqp-8* might be to enable or increase secretion by facilitating vesicle docking to the excretory canal lumen. The expression of *aqp-8* is activated by a secondary response to persist hyper-osmotic stress conditions. A complex regulatory network, consisting of the osmotic stress response, the response to protein damage and chemosensation by the amphid neurons, does regulate the expression of *aqp-8* (Figure 5).

## Materials and methods

### *C. elegans* strains

Standard techniques for *C. elegans* breeding were applied during this study (Stiernagle, 2006). NGM media plates seeded with the *E. coli* strain OP50 containing 50 mM NaCl were used to maintain *C. elegans*. All strains were raised and maintained at 20°C. Standard genetic crossing methods were used to create double mutants. Verification of the mutant genotypes was performed by standard PCR analysis and/or DNA sequencing.

The following strains were used: N2[BRISTOL]^*1^/AKR-14 +*/*+*;akaIs2*[K02G10.7(translational)::GFP +pCeh361]//LGI:*gpdh-1(ok1558)*//LGII: *osm-8(n1518)*/AKR-19*osm-8(n1518);akaIs2*[K02G10.7(translational)::GFP + pCeh361]//LGIII: *osm-7(n1515)*/LGX: aqp-8(ok2800)^*1^,aqp-8(tm1919)^*2^, *osm-11(n1604)*.

^*1^ The strains were obtained from the *C. elegans* Genetic Stock Center (University of Minnesota, USA). ^*2^ The strain was received from the National Bioresource Project for the Experimental Animal “Nematode *C. elegans*” (Japan).

### RNAi treatment

Single RNAi clones from the MRC library were used for RNAi treatment of *C. elegans* strains. Standard 10 cm NGM plates containing 1 mM ITPG (Carl Roth, Laborbedarf, Germany) were used for RNAi activation and seeded with 500 μl of RNAi bacteria (Rohlfing et al., 2011). *C. elegans* were hypochlorite treated, synchronized over night at 20°C and ~250 L1 animals were spotted onto each NGM plate. Animals were grown at 20°C for 4 days. At the first day of adulthood animals were screened for phenotypes or harvested for further analysis in M9 buffer, frozen in liquid nitrogen and stored at −80°C.

### Osmotic stress induction

Chronic hyper-osmotic stress conditions were induced by elevation of the NaCl concentration of the NGM (100, 150, 200 mM; Lamitina et al., 2004; Rohlfing et al., 2010, 2011). Approximately 250 hypochlorite treated and over night synchronized L1 animals were placed on the test plates and grown at 20°C. NGM plates (OP50 or RNAi bacteria) containing 50 mM NaCl were used as control conditions. Animals were harvested for further analysis at the first day of adulthood in M9 buffer, frozen in liquid nitrogen and stored at −80°C.

To induce acute hyper-osmotic stress conditions ~250 hypochlorite treated and synchronized animals were grown on standard 10 cm NGM (50 mM NaCl; OP50 or RNAi bacteria) at 20°C until first day of adulthood. The animals were harvested in M9 buffer and placed on 10 cm NGM plates (OP50 or RNAi bacteria) containing 200 mM NaCl. Again NGM plates containing 50 mM NaCl were used as control conditions. Animals were harvested in M9 buffer at the indicated time after exposure, frozen in liquid nitrogen and stored at −80°C until used for further analysis.

### Stress assays

Prior to the assays synchronized L1 animals were grown until first day of adulthood at 20°C on NGM plated with OP50 bacteria. To induce oxidative stress ~250 young adult animals were placed on 10 cm NGM OP50 plates containing 200 mM paraquat. After 1 h exposure animals were harvested for further analysis in M9 buffer, frozen in liquid nitrogen and stored at −80°C. To heat shock *C. elegans*, young adult animals were placed on 10 cm NGM OP50 plates pre-heated to 35°C. After half an hour animals were harvested in M9 buffer, frozen in liquid nitrogen and stored at -80°C until further use.

### Microscopy

Animals were anesthetized for microscopy in 10 mM sodium azide solved in M9 medium (Stiernagle, 2006) with the appropriate salt concentrations to maintain the test conditions. Microscopy analyses were performed using a laser-scanning microscope 510 (LSM510, Carl Zeiss, Jena, Germany). The obtained images were analyzed using the ZEN2008 software (Carl Zeiss, Jena, Germany). Animal length and the extent of AQP-8::GFP fluorescence were measured with the appropriate tools provided by the ZEN2008 software. Varicosities were defined as pearl-like extensions of the posterior excretory canal and counted manually.

### RNA preparation and quantitative PCR

RNA was purified from samples harvested from treated and untreated animals (see above) using TRIzol®;(Invitrogen) and the RNeasy RNA purification kit (Qiagen). The purified RNA was converted to cDNA (SensiFAST cDNA Synthesis Kit, Bioline). Quantitative PCR was carried out on an ABI7500fast qPCR System (Applied Biosysthems) using the SYBR green method (KAPA SYBR Fast qPCR Mastermix, PeqLab). The 7500 Software 2.0.1 (Applied Biosysthems) was used to calculate CT values and time fold changes as well as melting curve analysis of the product. The housekeeping gene *act-2* (T04C12.5, *C. elegans*) was used as normalizer. Primer pairs were designed to span an exon-exon boundary to minimize the risk of amplification of residual genomic DNA in the analyzed cDNA probes. All primer pairs were validated prior to use to secure copy number-dependent amplification and single products. Wildtype cDNA was analyzed for the presence of *aqp-8* isoforms by PCR, using standard PCR protocols (Taq DNA Polymerase, Roboklon; PCR cycler, Biometra). The PCR product was separated in 1% agarose gel and the result documented (Figure 2E). All primer pairs used in this study are listed in the dataset (Supplementary Table 2).

### Western blot

For the protein purification the samples harvested from treated and untreated animals (see above) were thawed on ice. Subsequent the samples were mixed with 300 μl HEPES-buffered saline (Stiernagle, 2006) containing proteinase inhibitors and grinded for 5 min on ice. The solution was centrifuged for 2 min at 4.000 rpm and the supernatant was used for further analysis. The protein concentration was determined using a standard Bradford assay (Bradford, 1976). SDS electrophoresis of the samples through 10% polyacrylamide gels, blotting onto nitrocellulose membranes and antibody detection were performed as described previously (Rohlfing et al., 2005). The detection of AQP-8::GFP was performed using 1:20,000 dilution of a mixture of two monoclonal Anti-GFP antibodies (Mouse IgG, clones 7.1 and 13.1, Cat. No. 11814460001, Roche) and a HRP-coupled goat anti-mouse (1:20,000, Jackson ImmunoResearch) as secondary antibody. Testing samples from N2[BRISTOL] animals not carrying *akaIs2*[aqp-8::GFP] results in the loss of the band labeled as specific signal (Supplementary Figure 1B). A SYPRO Ruby staining of the blotting membrane according to the manual (Molecular Probes, Invitrogen) was performed after blotting and before the antibody staining. The SYPRO Ruby staining of the total protein was used as loading control and normalizer to calculate the time fold change in protein abundance (Supplementary Figures 1A,C; Aldrige et al., 2008). The protein expression captured by western blot and SYPRO Ruby staining was analyzed using the densitometry measurement tool of the ZEN2008 software (Carl Zeiss, Jena, Germany).

### Genome wide RNAi screen

The genome wide RNAi screen was performed using the MRC feeding RNAi library and the supplement set (Source Bioscience, UK) covering ~87% of the *C. elegans* genes in 19,726 individual RNAi bacteria feeding strains. For the screen preparation the clones were stamped from the frozen stocks onto 96 well plates containing LB_*Carb*/*Tet*_ Agar and grown for 24 h at 37°C. From these plates 96 well plates containing 100 μl LB_*Carb*/*Tet*_ Medium were inoculated and grown for maximum 16 h at 37°C. Twenty-four well plates containing 2 ml NGM with 50 mM NaCl and 1 mM ITPG were seeded with 50 μl culture per well, each well representing another RNAi bacteria strain. The seeded plates were incubated over night at room temperature and stored at 4°C. Plates were used within 2 days after preparation. RNAi strains containing GFP RNAi vector and the empty RNAi vector L4440 were grown and seeded as described for the library strains. Those 24 well RNAi plates were used as control for the RNAi efficiency. AKR-14 +*/*+*;akaIs2*[K02G10.7(translational)::GFP + pCeh361] animals were used as test strain. Animals were hypochlorite treated and synchronized over night at 20°C. Approximately 25 synchronized L1 animals were seeded onto each well of the prepared RNAi plates and grown at 20°C for 4 days until first day of adulthood. Animals were screened for GFP expression using a fluorescence-equipped stereo dissecting microscope (Discovery V8, Zeiss, Germany). RNAi strains suppressing or enhancing the GFP expression of AKR-14 in the primary screen were further analyzed for effects on the AQP-8::GFP protein concentration by western blot analysis (see above). The inserts of effective RNAi suppressor or enhancer strains were verified by DNA sequencing.

### Statistical analysis

For statistical analysis and graphical presentation the program prism6 (GraphPad Software, LaJolla, USA) has been used. If not indicated otherwise mean and SE are plotted and Students *t*-tests were performed.

## Ethics statement

Only *C. elegans* strains and materials were used in this study. As invertebrate species the above mentioned requirements do not apply to *C. elegans*. By German and European law experiments on *C. elegans* do not require approval by an ethic committees. Although we tried to reduce and refine our experiments with live animals, e.g., salt exposure, to use the absolute minimum of animals.

## Author contributions

Conceptualization, Data Curation, Formal Analysis, Funding Acquisition, Methodology, Project Administration, Supervision, Validation, Visualization, and Writing—Original Draft Preparation: AR. Investigation: AR, CI, MJ, and JK. Resources: AR, and JK.

### Conflict of interest statement

The authors declare that the research was conducted in the absence of any commercial or financial relationships that could be construed as a potential conflict of interest.
