# Supplementary material for: Neuronal Chemosensation and Osmotic Stress Response Converge in the Regulation of aqp-8 in C. elegans
Source: Front Physiol. 2017 Jun 9;8:380. doi: 10.3389/fphys.2017.00380 (PMC5465262; doi:10.3389/fphys.2017.00380)
Supplement: Supplementary file 1 [file DataSheet1.docx]

Supplementary Material

Neuronal Chemosensation and Osmotic Stress Response Converge in the Regulation of *aqp-8* in *C. elegans*.

Carla Igual Gil, Mirko Jarius, Jens Peter von Kries and Anne-Katrin Rohlfing^*^

*** Correspondence:** Anne-Katrin Rohlfing / rohlfing@uni-potsdam.de

# Supplementary Figures and Tables

## Supplementary Figures


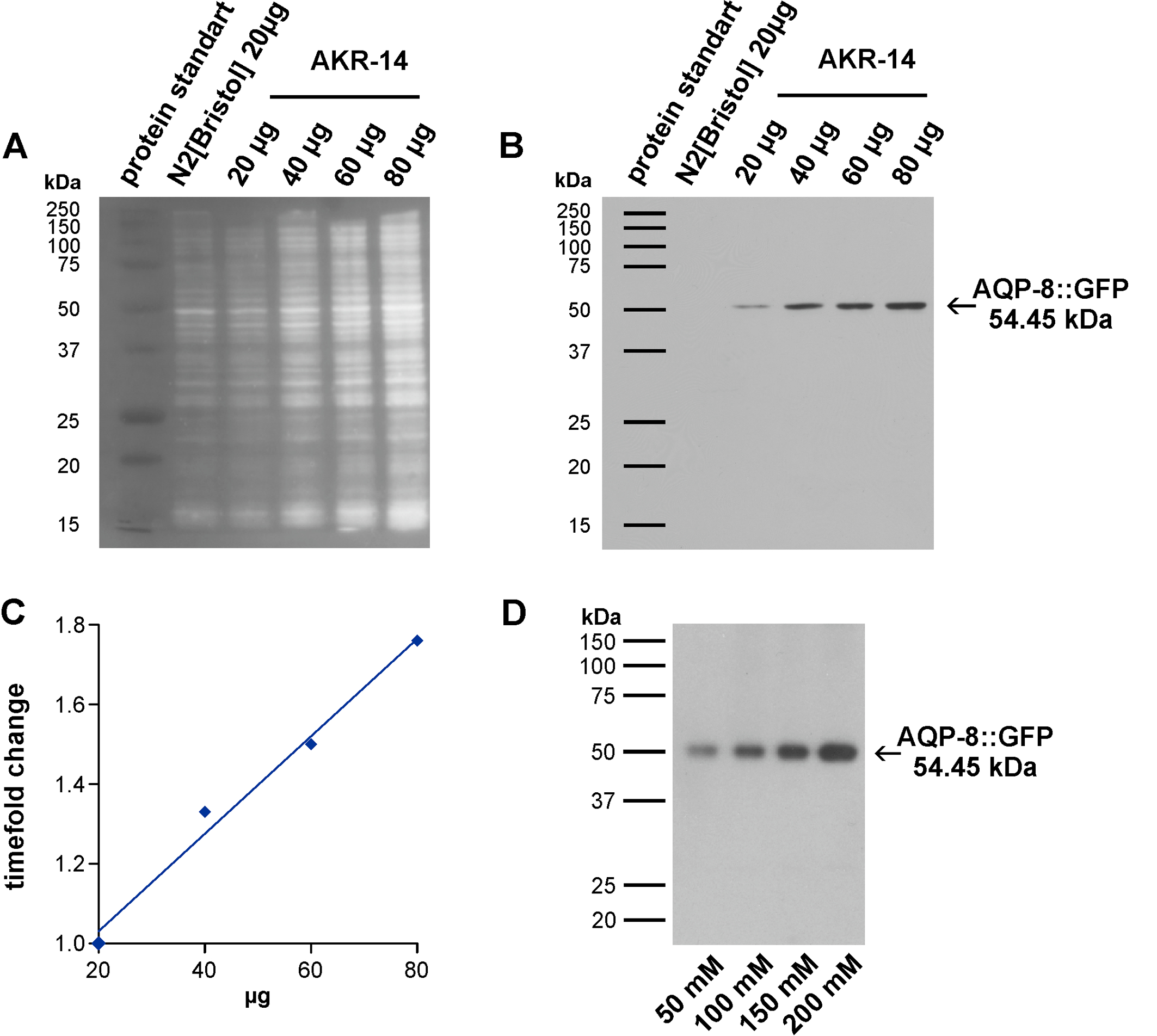


**Supplementary Figure 1. Verification of SYPRO Ruby staining and GFP antibody for use in quantitative western blot analysis** (A) SYPRO Ruby staining of the PVDF membrane used for GFP antibody test western blot (S1 Supp Fig 1B). Wild type (20 µg) and AKR14 (20 µg) show the same fluorescence intensity. The SYPRO Ruby does signal increases according to protein loaded (lane 3-6). (B) Western blot testing the GFP antibody used to blot AQP-8::GFP. The AQP-8::GFP Signal is visible at approximately 54.45 kDa, the calculated weight of the fusion protein. The signal increases according to protein concentration loaded (20 -80 µg) in AKR-14 animals carrying the transgene but is absent in the wild type control lane. (C) Plotted is the SYPRO Ruby fluorescence signal of each AKR-14 lane of the PFDV membrane used for GFP antibody testing, representing the amount of protein loaded. The linear regression and coefficient of determination (R^2^ = 0.9979) were calculated. (D) Sample western blot of the increase in AQP-8::GFP signal under various chronic stress conditions.

**
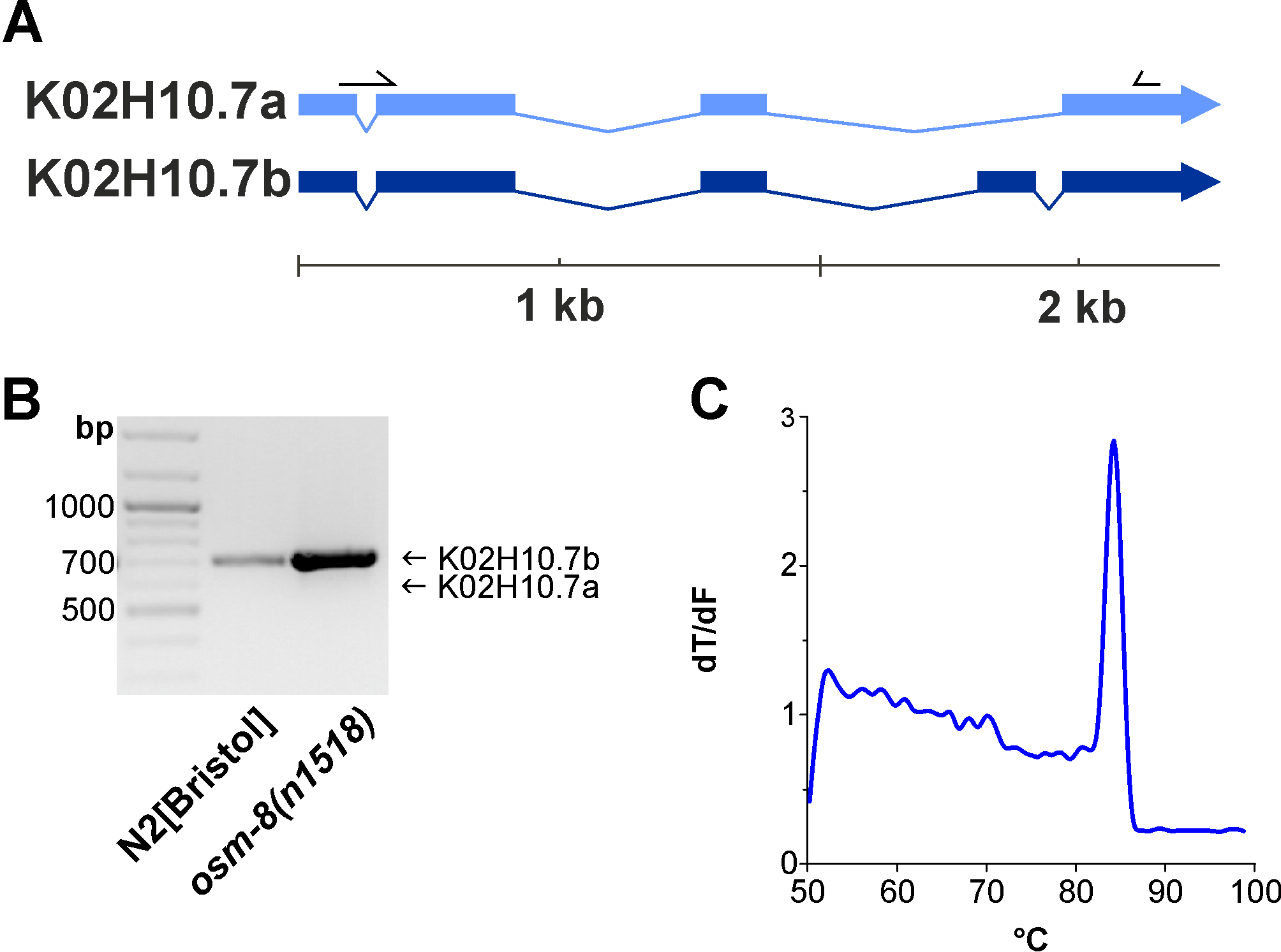
**

**Supplementary Figure 2. Isoform analysis.** (A) Graphical presentation of the *aqp-8* isoforms K02H10.7a and K02H10.7a. Black arrows mark the position of the primer pair used for isoform analysis. (B) 1% agarose gel showing two *aqp-8* PCR samples. Only the isoform b (708 bp) is present in these samples from wild type animals and *osm-8(n1518).* The stronger expression of *aqp-8* in an *n1518* background is also reflected in gel. (C)Melting curve analysis of *aqp-8* isoforms qPCR. Plotted is the mean of four technical replicates.


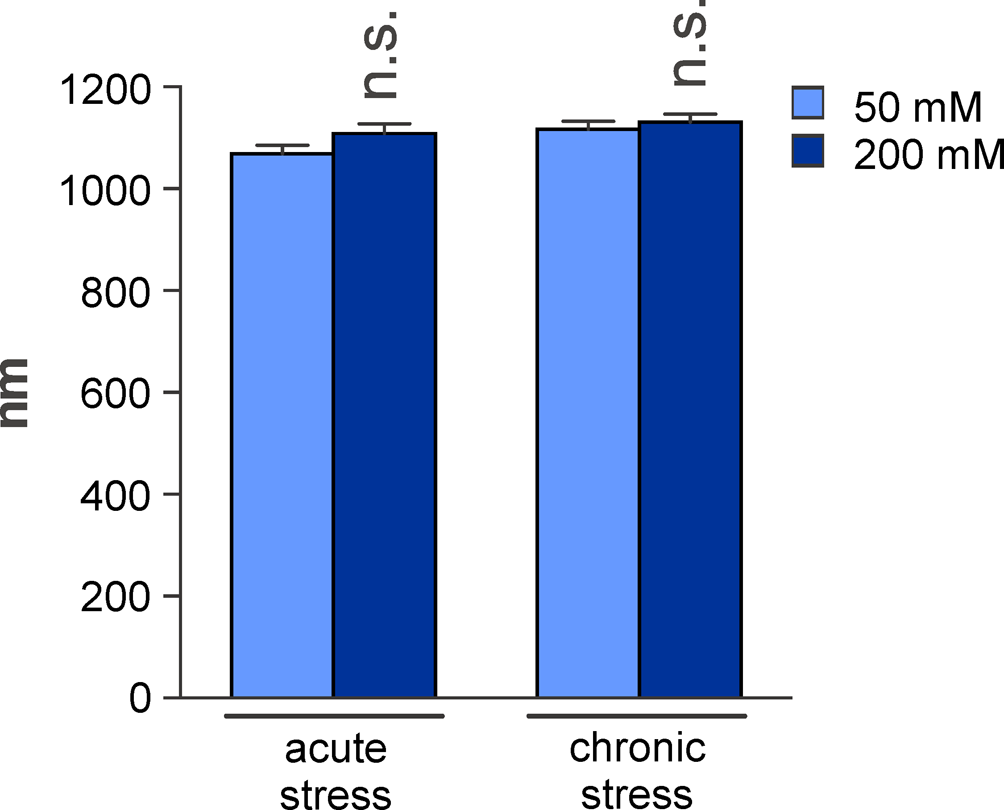


**Supplementary Figure 3. Body length of test animals.** Comparison of the body length of the test animals after 8h acute and chronic 200 mM NaCl stress conditions compared to 50 mM NaCl control conditions. Student’s t-tests were performed relative to control conditions; N>16. All qPCR expression levels were normalized to *act-2* (actin). All values are indicated relative to control conditions (set to 1). * p< 0.05; ** p < 0.01; *** p<0.001; n.s. - not significant.

**
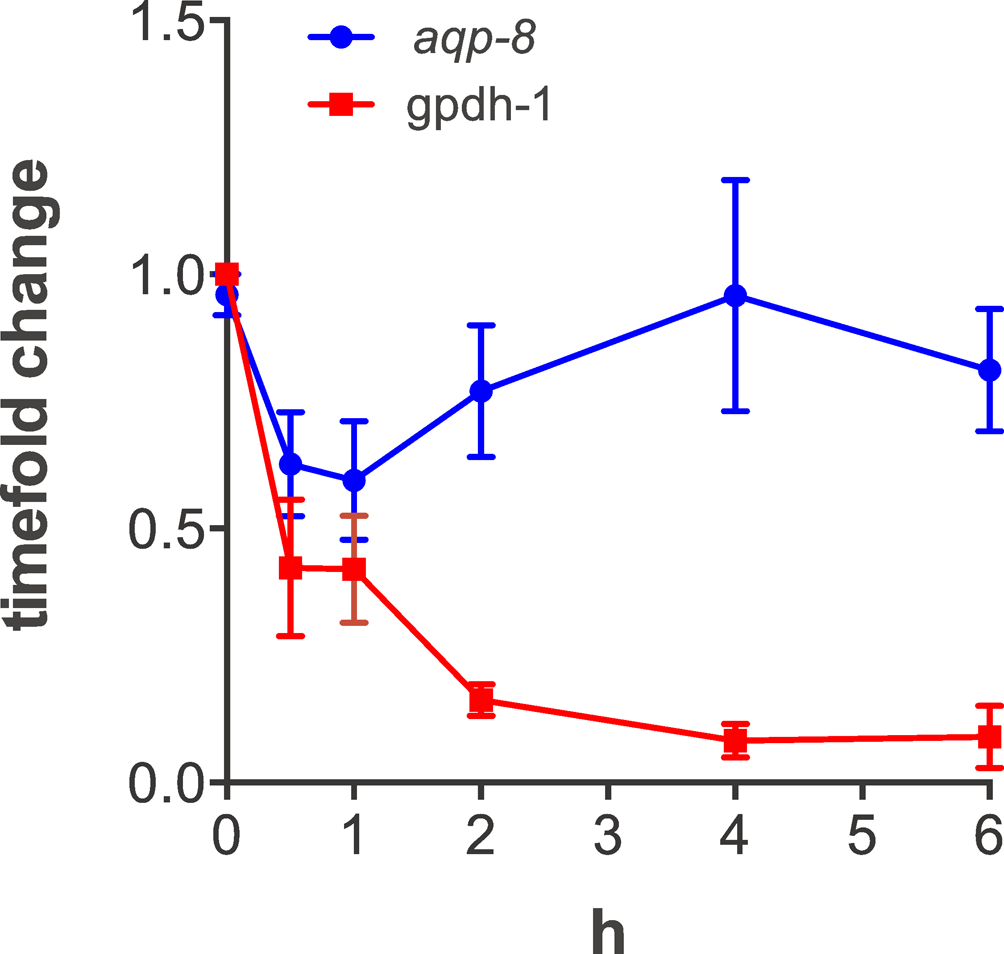
**

**Supplementary Figure 4. Control values for *gpdh-1* and *aqp-8* mRNA expression over time.** *gpdh-1* and *aqp-8* mRNA expression levels on day one of adulthood under control conditions (50 mM NaCl).


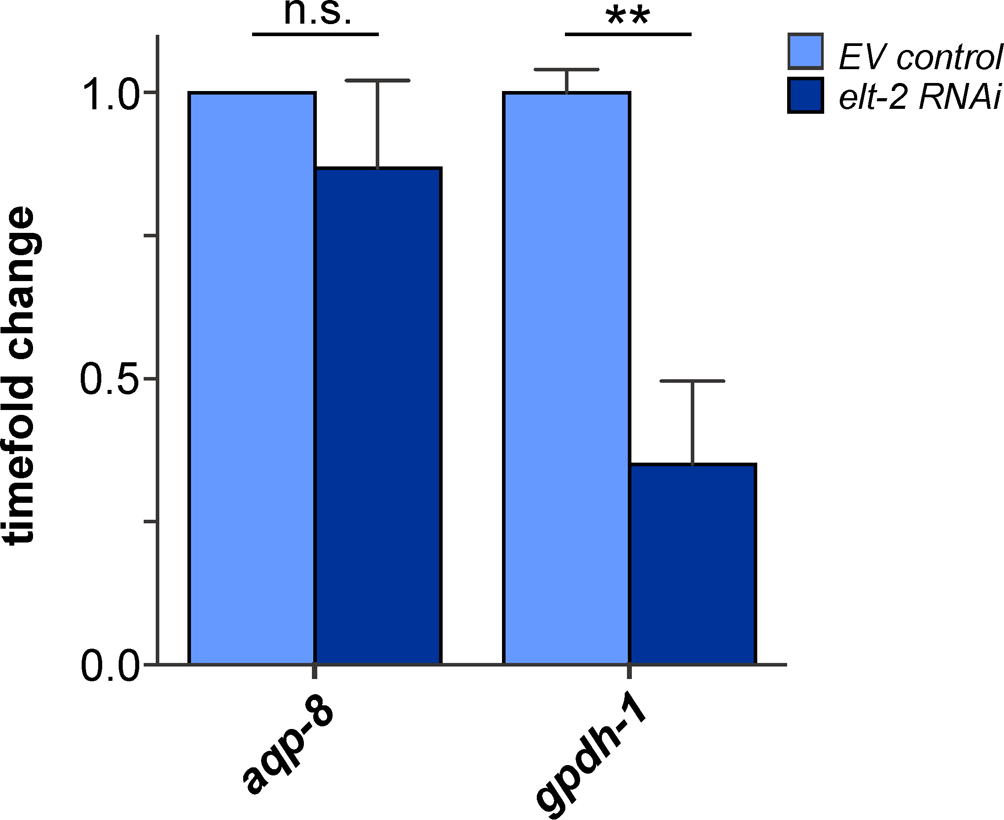


**Supplementary Figure 5. Effects of *elt-2* RNAi on *aqp-8* expression in an *osm-8(n1518)* background.** RNAi effect on *aqp-8* and *gpdh-1* mRNA expression levels in an *osm-8(n1518)* background. Student’s t-tests were performed between empty vector controls qPCR result (L4440; light blue) and *elt-2* RNAi result (dark blue); N>4.

## Supplementary Table


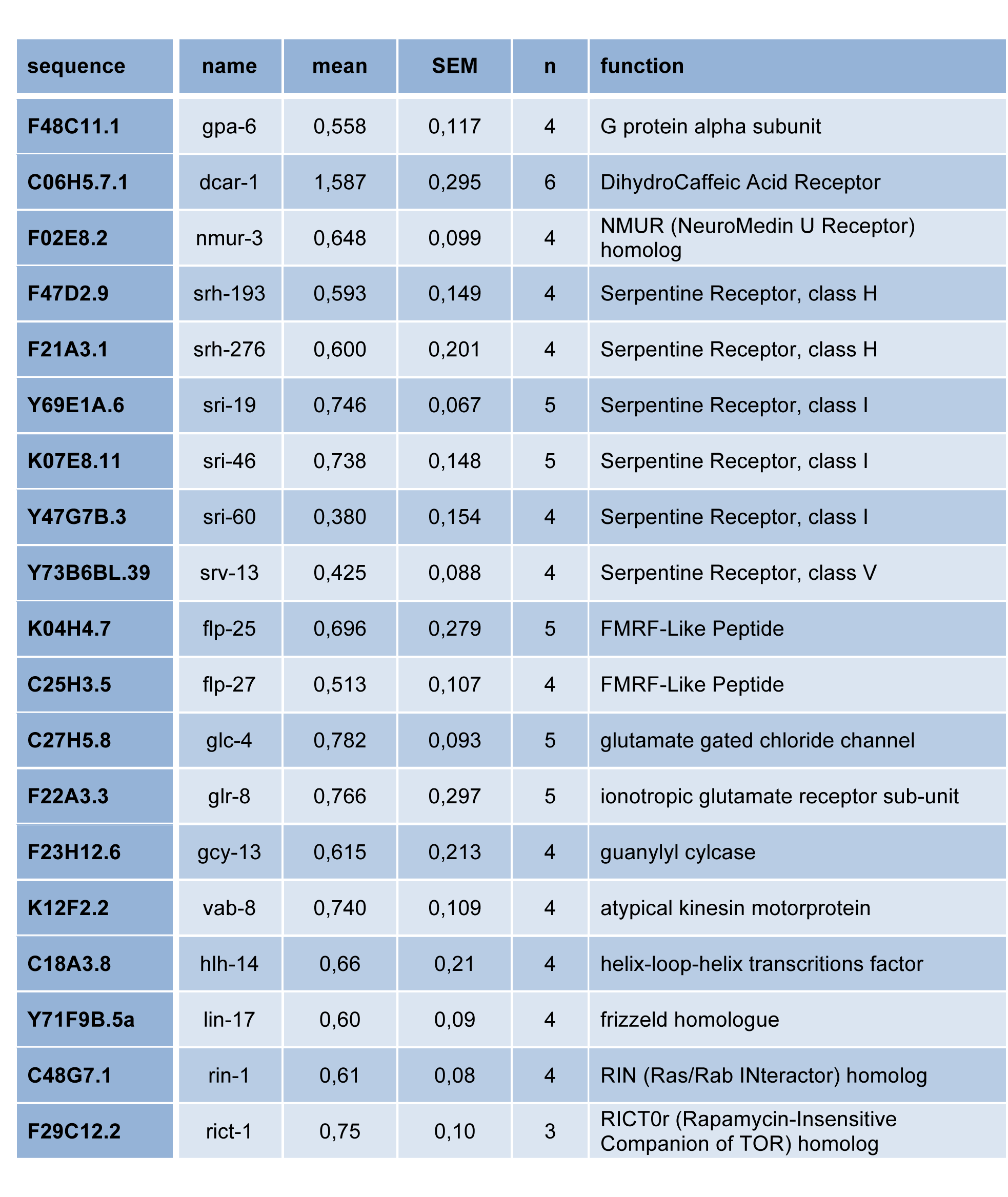


**Supplementary Table 1. Possible regulators of *aqp-8* expression.** List of genes, derived from the performed genome-wide RNAi screen, connected to neuronal functions that are affecting *aqp-8* expression. For each gene the results of the western blot analysis performed to verify the primary screen are shown: relative expression level of AQP-8::GFP compared to control conditions (mean), standard error of mean (SEM) and replicate number (n).


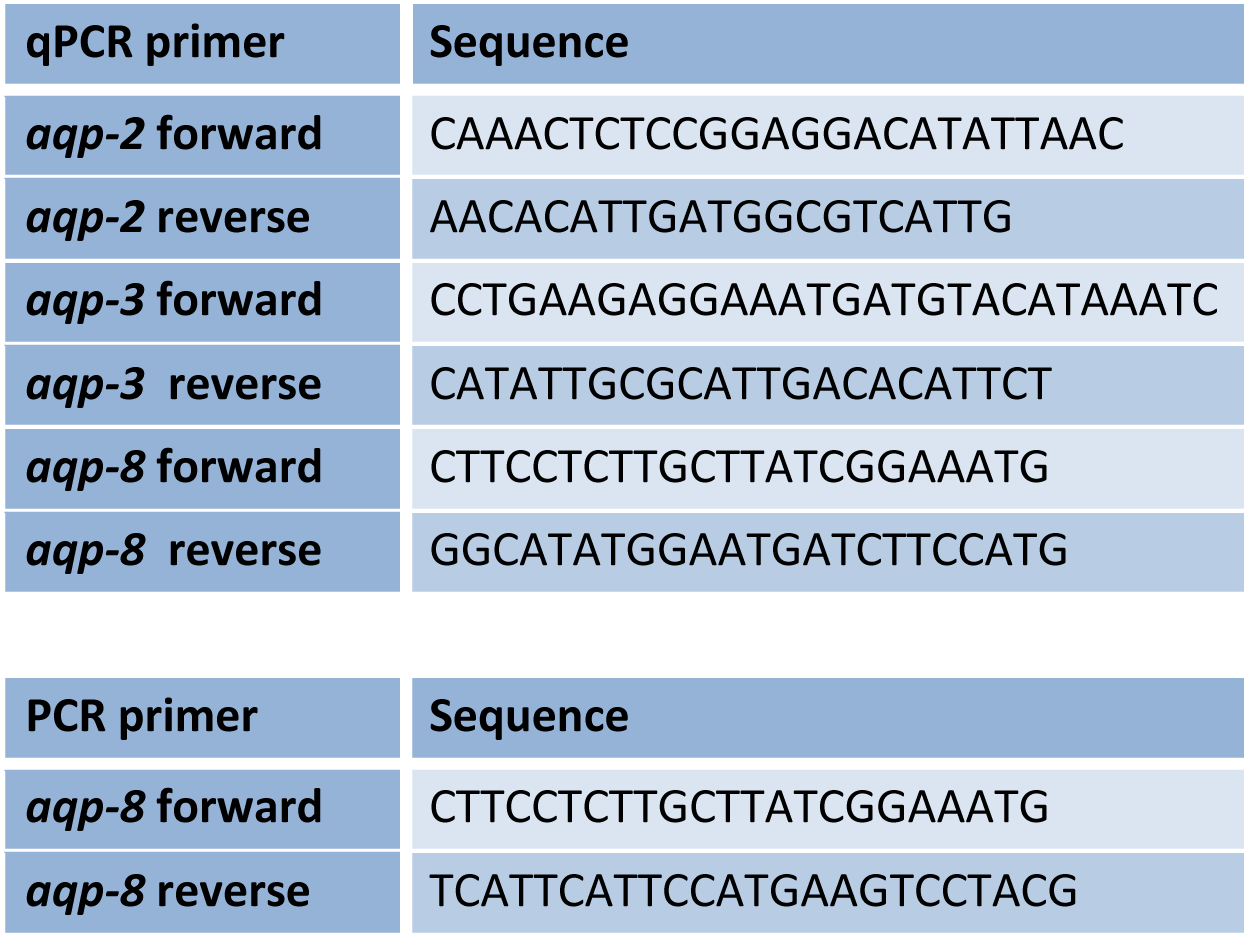


**Supplementary Table 2. List of primers used in this publication.**
